# Comparison of Hydroxychloride Versus Oxide and Sulfate Sources of Manganese, Zinc, and Copper in Rearing Diets on Pullet Growth, Tibia Traits, and Egg Production and Eggshell Quality in ISA Brown Hens up to 50 Weeks

**DOI:** 10.3390/ani14243581

**Published:** 2024-12-11

**Authors:** Clara Alfonso-Carrillo, Reza Akbari Moghaddam Kakhki, Ana Isabel Garcia-Ruiz

**Affiliations:** Poultry Research Centre, Trouw Nutrition R&D, El Viso de San Juan, 45950 Toledo, Spain; c.alfonso@trouwnutrition.com (C.A.-C.); ai.garcia@trouwnutrition.com (A.I.G.-R.)

**Keywords:** trace mineral, carryover effect, laying hen, eggshell, pullet growth

## Abstract

(1) Background: this study compared hydroxychloride and traditional oxide/sulfate sources of zinc (Zn), manganese (Mn), and copper (Cu) in ISA Brown pullet diets, focusing on growth, tibia strength, egg production, and eggshell quality. (2) Methods: in total, 120 pullets were divided into two groups, each with six replicates of 10 birds, receiving diets with hydroxychloride or oxide/sulfate sources of Mn, Zn, and Cu (65, 50, and 5 mg/kg, respectively) during rearing. At 16 weeks, 18 pullets per group transitioned to individual cages and were fed a standard diet with Mn-oxide, Zn-oxide, and Cu-sulfate until 50 weeks. (3) Results: pullets fed hydroxychloride minerals exhibited a higher feed conversion ratio (*p* = 0.023) and a trend toward higher average daily feed intake (*p* = 0.059) compared to those on oxide/sulfate sources during the rearing phase. During the laying phase, while no significant differences were observed in egg production until 24 weeks, hens previously fed hydroxychloride minerals demonstrated increased average daily feed intake and egg weight from 25 to 50 weeks of age (*p* < 0.05). (4) Conclusions: rearing diets supplemented with hydroxychloride minerals influenced feed intake and efficiency, with carryover effects that enhanced laying-phase performance.

## Simple Summary

The effects of different forms of essential minerals—zinc, manganese, and copper—on pullet growth and egg quality were investigated. Typically, these minerals are provided as oxides and sulfates, but newer forms, such as hydroxychlorides, are considered more efficient and environmentally friendly. During the rearing phase, pullets were fed diets containing either traditional mineral sources or hydroxychlorides, and their performance was monitored as they matured into egg-laying hens. Pullets receiving hydroxychloride minerals exhibited higher feed intake. These effects persisted into the laying period, with hens from the hydroxychloride group consuming more feed and producing heavier eggs. These findings suggest that the mineral form in early diets can have lasting impacts on laying performance, providing insights for optimizing egg production and promoting sustainable farming practices.

## 1. Introduction

Trace minerals such as zinc (Zn), manganese (Mn), and copper (Cu) play critical roles in bone formation and eggshell quality [1,2]. Zn supports bone and cartilage integrity by facilitating collagen formation and hydroxyapatite crystallization [3], while Mn contributes to bone development by activating enzymes involved in synthesizing mucopolysaccharides in bone cartilage [4,5]. Cu is essential for maintaining bone tensile strength by aiding in the cross-linking of elastin and collagen, ensuring structural integrity [6].

In addition to their roles in bone health, these minerals are integral to eggshell formation. Mn activates enzymes responsible for synthesizing glycosaminoglycans and glycoproteins, which form the organic matrix of the eggshell [4,7]. Zn serves as a cofactor for carbonic anhydrase, an enzyme critical for eggshell calcification, while Cu is a key component of lysyl oxidase, which facilitates collagen cross-linking in the eggshell membrane [8].

With the increasing demand for eggs, maintaining peak bone mineral density before laying begins is crucial for ensuring eggshell quality, as approximately 40% of the calcium needed for eggshell formation comes from bones [9]. Eggshell quality is essential for minimizing economic losses, as cracked eggs account for 3–5% of production, depending on the housing system [10].

Traditionally, poultry diets have included inorganic salts (sulfates, oxides) to supply Cu, Mn, and Zn minerals. The growing demand for ingredients with higher bioavailability has increased interest in hydroxychloride trace minerals. These minerals offer advantages over traditional inorganic salts due to their stronger covalent bonds, better solubility at low pH, and enhanced absorption rates [11]. Unlike traditional inorganic forms, hydroxychlorides do not form indigestible complexes with dietary components, improving mineral bioavailability [12].

Despite extensive research on Zn, Mn, and Cu trace minerals [13,14], the comparative effects of hydroxychloride sources versus other inorganic forms during the rearing phase remain underexplored. A previous study evaluated the efficacy of hydroxychloride and organic sources of these minerals in the rearing diets of Lohmann Brown pullets, revealing higher average daily feed intake (ADFI) and egg mass (EM) during the laying cycle (25–50 weeks of age) in the organic group [15]. Building on this research, the present study aimed to compare different inorganic sources of Mn, Zn, and Cu—including hydroxychloride versus traditional oxide and sulfate forms in the rearing diets—in terms of pullet performance, tibia quality, and potential carryover effects on production performance and eggshell quality during the laying phase.

## 2. Materials and Methods

### 2.1. Animal Management

This study was approved by the Animal Welfare Committee of Trouw Nutrition R&D Poultry Research (internal animal welfare project 13-2021), ensuring that all procedures complied with ethical standards for the care and use of birds in experimental studies [16]. A total of 120 ISA Brown day-old pullets were obtained from a commercial hatchery and housed in group cages at the Trouw Nutrition Poultry Research Centre in El Viso De San Juan, Toledo, Spain.

Upon arrival, the room temperature was set to 35 °C and was gradually reduced to 20 °C by day 35, where it was maintained until the experiment concluded. Pullets received 24 h of light at 40 lux during the first week, which was gradually reduced to 9 h at 6 lux by week 7. This lighting schedule was maintained until week 16, after which it was increased to 14 h for the remainder of the laying phase.

### 2.2. Experimental Treatments

During the rearing phase (0–16 weeks), the pullets were randomly assigned to one of two dietary treatment groups, each consisting of six replicates with 10 birds per replicate. Diets were formulated to meet or exceed the nutrient requirements defined by CVB (2018) [17] and were prepared from the same batch of raw materials (Table 1). The target supplementation levels for Mn, Zn, and Cu were 65, 50, and 5 mg/kg, respectively, as recommended by Leeson and Summers (2009) [18]. Experimental diets, provided in crumble form, were supplemented with a trace mineral premix without Mn, Zn, or Cu. For the control group, minerals were supplied as feed-grade Mn-oxide (62%), Zn-oxide (72%), and Cu-sulfate (25%). For the treatment group, minerals were supplied as hydroxychloride forms (Intellibond^®^, Trouw Nutrition, Tilburg, The Netherlands).

### 2.3. Laying Phase

At 16 weeks, 18 birds from each treatment group were transferred to individual cages in a laying facility. They were fed standard laying-hen diets supplemented with Mn-oxide, Zn-oxide, and Cu-sulfate in mash form to evaluate the carryover effects of rearing-phase treatments. From the start of lay to 25 weeks, the birds received a commercial mash diet (Nanta, Grinon, Spain) containing 2750 Kcal/kg AME, 3.3 g/kg digestible phosphorus, and 166.8 g/kg crude protein, as well as 40.2 g/kg calcium and 6.9 g/kg phosphorus (analyzed values). From 25 to 50 weeks, the diet was adjusted to provide 2800 Kcal/kg AME, 3.1 g/kg digestible phosphorus, with analyzed values of 155.6 g/kg crude protein, 45.5 g/kg calcium, and 6.7 g/kg phosphorus.

### 2.4. Feed Analysis

Dietary samples were analyzed for dry matter (930.15), crude protein (968.06), ash (942.05), crude fiber (962.09), and ether extract (920.39) using AOAC methods (Table 1) [19]. Calcium and phosphorus were analyzed spectrophotometrically at MasterLab Trouw Nutrition (Madrid, Spain), with calcium determined using Gitelman’s method [20] and phosphorus quantified using the method of Murphy and Riley [21]. Mn, Zn, and Cu concentrations were measured via inductively coupled plasma mass spectrometry according to the standard 17,053 of the Spanish Association for Standardization and Certification (2018) [22].

### 2.5. Measurments and Sample Analysis

The ADFI and body weight (BW) were recorded at the end of each feeding phase. Hen-day egg production (HDEP) and egg weight (EW) were monitored daily. Feed conversion ratio (FCR) was calculated as the feed intake to BW ratio for the rearing phase (0–16 weeks) and as the ADFI to EM (HDEP × saleable egg weight) ratio for the laying phase (18–50 weeks).

Six birds per treatments group were euthanized by cervical dislocation at 16 weeks (within ±95% of the unit’s average BW), and tibias were collected for analysis. Right tibias were dried at 103 °C for 18 h and ashed at 550 °C for 12 h [15]. The left tibias were analyzed at the Universidad de Granada for water, organic matter, carbonate, and phosphate content using thermogravimetric analysis. Approximately 25 mg of powdered bone from the tibia cortex was placed in a crucible and processed using a METTLER-TOLEDO thermogravimetric system (model TGA/DSC1). The analysis, performed at a heating rate of 20 °C/min, recorded thermogravimetric analysis curves from room temperature to 950 °C [23]. The percentages of organic matter, carbonate, and phosphate were expressed relative to dry matter.

From week 25 onwards, four intact eggs per cage were sampled every 4 weeks to measure egg component yields and eggshell quality parameters, including breaking strength, shell weight, and shell thickness. Eggshells were dried, and eggshell percentage was calculated relative to EW. Eggshell breaking strength and thickness were measured using a texture analyzer (TA.XT plus100C, Stable Micro Systems, Surrey, UK) [15]. Shell weight per unit surface area (SWUSA) was calculated by dividing eggshell weight (mg) by surface area [24]. Albumen weight was determined by subtracting yolk and shell weights from EW.

### 2.6. Statistical Analysis

The normality of the data was assessed using the PROC UNIVARIATE procedure in SAS^®^ 9.4 (SAS, Cary, NC, USA), with the Shapiro–Wilk test used as a formal test for normality. The data were confirmed to follow a normal distribution, and any identified outliers (mean ± 3.0 SD) were evaluated using the INFLUENCE statement within the MIXED procedure of SAS^®^ (SAS, Cary, NC, USA). During the rearing phase, cages were considered the experimental units, while individual birds served as experimental units during the laying phase. Production and eggshell quality data were analyzed as repeated measures, while other parameters were analyzed for main treatment effects using the MIXED procedure of SAS^®^. Differences between least-square means were assessed using the Dunnett test. Statistical significance was set at *p* ≤ 0.05, with trends reported for 0.05 < *p* ≤ 0.10.

## 3. Results

### 3.1. Growth Performance

During the starter and developer phases, no significant differences were observed in ADFI, BW, or FCR between pullets fed hydroxychloride and those fed oxide/sulfate sources of Mn, Zn, and Cu (*p* > 0.05; Table 2). However, during the grower phase, ADFI and FCR were significantly higher in the hydroxychloride group compared to the oxide/sulfate group (*p* < 0.05), while BW remained unaffected (*p* > 0.05).

Overall, ADFI tended to be higher in pullets receiving hydroxychloride minerals compared to those on oxide/sulfate minerals (*p* = 0.059), and FCR was significantly greater in the hydroxychloride group (*p* = 0.023).

### 3.2. Egg Production Performance

During the laying phase, no significant interaction between treatments and week was observed (*p* > 0.05; Table 3). From 18 to 24 weeks of age, no differences were found in egg production parameters, including ADFI, HDEP, EW, EM, or FCR, as well as BW at 25 weeks of age (*p* > 0.05).

Between 25 and 37 weeks of age, hens previously fed hydroxychloride sources during the rearing phase exhibited significantly higher ADFI, EW, and EM compared to those fed oxide/sulfate sources (*p* < 0.05; Table 3). However, no significant differences were observed in HDEP or FCR between the treatments.

From 38 to 50 weeks of age, hens previously fed hydroxychloride sources showed significantly higher ADFI and EW compared to those fed oxide/sulfate sources (*p* < 0.05; Table 3). Additionally, there was a tendency for hens fed hydroxychloride sources to exhibit higher EM and FCR compared to those on oxide/sulfate sources (*p* < 0.10). No differences were observed in HDEP or BW among the treatments during this period.

### 3.3. Egg Quality

There was no significant interaction between treatments and week in egg quality parameters. From 25 to 37 weeks of age, hens fed hydroxychloride sources during the rearing phase tended to have a higher yolk percentage compared to those fed oxide/sulfate sources (Table 4). However, no significant differences were observed in albumen percentage, eggshell percentage, eggshell breaking strength, eggshell thickness, or shell weight per unit surface area (*p* > 0.05).

From 38 to 50 weeks of age, hens previously fed hydroxychloride sources during rearing exhibited a lower eggshell percentage compared to those in the oxide/sulfate group (*p* = 0.039; Table 4). No significant differences were observed in the other egg quality parameters during this period (*p* > 0.05).

### 3.4. Tibia Quality

Tibia quality parameters, including dry weight, ash content, ash percentage, breaking strength, and the composition of the cortical bone at 16 weeks of age, showed no significant differences between the two mineral source groups (*p* > 0.05; Table 5).

At 50 weeks of age, birds fed hydroxychloride sources during the rearing phase exhibited higher tibia dry weight compared to those in the oxide/sulfate group (*p* = 0.014). However, no significant differences were observed in ash content, ash percentage, tibia breaking strength, or the composition of the cortical and medullary parts of the tibia among the treatment groups (*p* > 0.05; Table 5).

## 4. Discussion

The discrepancies between calculated and analyzed levels of Mn, Zn, and Cu in the diets, with recovery rates of 79 ± 6.6%, 72 ± 3.1%, and 47 ± 6.8%, respectively, highlight the variability introduced by natural plant-based mineral content. This variability is influenced by factors such as soil composition, agricultural practices, and processing methods, which can result in differences between theoretical and actual mineral concentrations in formulated diets [25].

In the current study, while pullets fed hydroxychloride sources showed higher ADFI compared to the oxide/sulfate group during the rearing phase, no differences in BW were observed. These findings align with previous research by Olukosi et al. (2018), who reported no significant effects on BW or ADFI in broilers when substituting sulfate-based Zn (80 mg/kg) and Cu (15 mg/kg) with hydroxychloride-based sources [26]. Similarly, studies on Lohmann Brown pullets found no significant differences in BW, ADFI, or FCR when rearing diets contained hydroxychloride sources of Mn, Zn, and Cu compared to organic sources [15]. Palanisamy et al. (2023) also observed no differences in ADFI or egg production parameters in White Leghorn hens fed hydroxychloride-bound Mn, Zn, and Cu (60, 60, and 15 mg/kg, respectively) compared to hens fed sulfate- and oxide-based mineral sources (80, 80, and 15 mg/kg, respectively) [14].

The higher ADFI observed in pullets fed hydroxychloride sources may be explained by the potential improvement in palatability. Hydroxychloride minerals are less soluble and may reduce the “metallic taste” associated with sulfate-based sources, encouraging higher feed intake [27]. This effect has also been reported in other species, such as beef calves, where hydroxychloride minerals led to increased feed intake compared to sulfate sources [27]. Additionally, Macelline et al. (2024) found positive correlations between trace mineral inclusion levels (Zn, Cu, and Mn) and ADFI during the finisher phase of modern broiler chickens, suggesting that hydroxychloride minerals may enhance nutrient bioavailability and absorption [28].

The relationship between trace minerals and feed intake remains an area of limited understanding in poultry. While Mn’s and Cu’s roles in feed intake regulation are not well-defined, Zn deficiency has been shown to reduce ADFI in broilers [29]. In pigs, dietary Zn enhances feed intake by stimulating the orexigenic effects of ghrelin, a hormone that crosses the blood–brain barrier and stimulates appetite through orexigenic neurons [30].

The higher ADFI observed during the rearing phase also appeared to have a long-term effect on ADFI and egg production performance during the laying phase when all hens were fed oxide/sulfate sources of Mn, Zn, and Cu. While there is limited documentation on the long-term effects of rearing diets on laying performance, previous research on Lohmann Brown pullets fed organic sources of Mn, Zn, and Cu reported higher ADFI and EM compared to pullets fed hydroxychloride sources during rearing, indicating a potential carryover effect [15].

Studies evaluating the effects of trace mineral sources in laying diets have shown inconsistent results. For example, Olukosi et al. (2019) observed no significant effects on HDEP, FCR, EM, eggshell thickness, or eggshell percentage when hydroxychloride sources replaced sulfate forms in 24-week-old Lohmann Brown hens, but they reported reductions in EW and cracked egg percentages [31]. In contrast, Jiang et al. (2021) found no significant differences in HDEP, EW, FCR, or broken egg percentages when replacing sulfate-based trace minerals with hydroxychloride sources [32]. Furthermore, long-term studies replacing inorganic Zn (30 mg/kg) and Mn (50 mg/kg) with organic sources reported no significant impact on egg production performance from 20 to 70 weeks of age [33].

Regarding eggshell quality, substituting inorganic sources of Mn, Zn, and Cu with hydroxychloride sources has been associated with improvements in previous studies [32]. Broiler breeders also showed enhanced eggshell quality when fed a blend of 80% hydroxychloride and 20% organic sources of these minerals compared to 100% organic sources [1].

The roles of Mn, Zn, and Cu in enzymes such as carbonic anhydrase, lysyl oxidase, and those involved in glycosaminoglycan and glycoprotein synthesis are critical for eggshell formation [4,7,8]. However, the lack of significant effects on eggshell quality in this study may be attributed to the transition from hydroxychloride or organic sources in the rearing diets to sulfate/oxide sources in the laying diets. Additionally, the minimal impact of Mn, Zn, and Cu sources on bone development during the rearing phase may have influenced eggshell outcomes.

Contrary to our findings on tibia quality, which showed no significant differences among treatments, Nguyen et al. (2021) demonstrated that diets lacking Zn negatively affected tibia development in broilers, with tibia breaking strength improving linearly with increasing levels of zinc hydroxychloride [34]. Similarly, Sadr et al. (2024) reported no effects on tibia breaking strength or ash percentage when supplementing broiler diets with various ratios of hydroxychloride and organic Zn at 80 mg/kg [35].

Olukosi et al. (2018) also observed no effect on tibia ash percentage in broilers fed hydroxychloride-based Zn (80 mg/kg) and Cu (15 mg/kg) compared to sulfate-based sources [26]. Additionally, Nguyen et al. (2020) found no variations in tibia ash content or breaking strength in broilers fed Cu from either sulfate or hydroxychloride sources at levels of 15 or 200 mg/kg [13]. Similarly, research on Lohmann Brown pullets reported no significant differences in tibia parameters when comparing hydroxychloride and organic sources in rearing diets [15].

The lack of differences in tibia quality in this study may be due to the inclusion levels of Mn, Zn, and Cu, which, while slightly lower than breeder recommendations (85 mg/kg Mn, 80 mg/kg Zn, and 10 mg/kg Cu) [36], were sufficient to meet the actual growth requirements of pullets. This may have masked potential treatment effects. Additionally, the higher average BW (1650 g) observed in the current study, which exceeded the breeder’s recommended range of 1409–1481 g, likely reflects the nutritional adequacy of the diets rather than differences in mineral sources. This higher BW can be attributed to the consumption of crumble diets, which may have supported better nutrient intake and growth.

## 5. Conclusions

This study demonstrated that the source of Mn, Zn, and Cu in rearing diets significantly influenced ADFI and FCR during the rearing phase, with lasting effects on ADFI and egg production performance up to 50 weeks of age. However, the tested levels of trace mineral sources did not impact BW, eggshell quality, or tibia quality. These findings underscore the potential influence of mineral sources, both directly and through carryover effects, on ADFI. Future research should focus on investigating the underlying mechanisms that regulate feed intake, ADFI, and water intake, particularly under heat stress conditions where feed intake typically declines. Such studies are essential for optimizing poultry production and improving long-term performance.

## Figures and Tables

**Table 1:** The experimental diets with different manganese, zinc, and copper sources during the rearing phase.

Items	Starter (1–3 Week)		Grower (4–10 Week)		Developer (11–16 Week)	
	Oxide/Sulfate	Hydroxychloride	Oxide/Sulfate	Hydroxychloride	Oxide/Sulfate	Hydroxychloride
Ingredient, g/kg						
Maize	400.00	400.00	400.00	400.00	612.00	612.00
Wheat	226.00	226.00	108.00	108.00	60.00	60.00
Soybean meal, CP = 480 g/kg	286.00	286.00	250.00	250.00	65.00	65.00
Sunflower meal, CP = 310 g/kg					120.00	120.00
Barley			150.00	150.00		
Barley straw					41.00	41.00
Potato protein					43.00	43.00
Wheat bran			30.00	30.00		
Soybean oil	17.40	17.40	5.00	5.00	5.00	5.00
Oat hulls	30.00	30.00	30.00	30.00	30.00	30.00
Calcium carbonate	16.16	16.22	12.58	12.64	7.91	8.10
Monocalcium phosphate	4.81	4.81	2.25	2.25	2.12	2.12
Filler (magnesium silicate)	4.11	4.05	1.35	1.29	4.07	3.89
Sodium chloride	2.79	2.79	3.03	3.03	2.52	2.51
Sodium bicarbonate	2.29	2.29	0.47	0.47	0.85	0.85
Dl-Methionine 99%	2.23	2.23	1.22	1.22	0.16	0.16
L-Lysine HCl 98%	1.39	1.39	0.10	0.10	0.37	0.37
AXTRA XAP ^1^	1.00	1.00	1.00	1.00	1.00	1.00
L-Threonine 98%	0.82	0.82				
Premix (oxide and sulfate) ^2,3^	5.00		5.00		5.00	
Premix (hydroxychloride) ^2,3^		5.00		5.00		5.00
Calculated nutrients (analyzed nutrients ^4^)						
AME, Kcal/kg	2850	2850	2750	2750	2700	2700
Crude protein, g/kg	190.45 (196.5)	190.45 (194.8)	177.54 (172.0)	177.54 (177.5)	150.00 (155.3)	150.00 (148.3)
Crude fiber, g/kg	34.18 (31.9)	34.18 (31.0)	38.37 (34.8)	38.37 (39.0)	70.93 (66.3)	70.93 (67.4)
Crude fat, g/kg	43.98 (36.0)	43.98 (35.0)	32.34 (23.0)	32.34 (22.0)	35.46 (27.5)	35.46 (23.5)
Dig lysine, g/kg ^5^	9.50	9.50	7.80	7.80	6.20	6.20
Dig methionine, g/kg	4.98	4.98	3.57	3.57	2.80	2.80
Dig methionine + cystine, g/kg	7.70	7.70	6.01	6.01	4.84	4.84
Dig threonine, g/kg	6.84	6.84	5.30	5.30	4.20	4.20
Calcium, g/kg	9.00 (7.6)	9.00 (7.7)	7.60 (5.5)	7.60 (5.5)	6.70 (5.2)	6.70 (5.5)
Digestible phosphorus, g/kg	3.80	3.80	3.30	3.30	2.90	2.90
Total phosphorus, g/kg	4.53 (4.3)	4.53 (4.1)	3.99 (3.7)	3.99 (3.9)	3.44 (2.6)	3.44 (2.9)
Sodium, g/kg	1.80	1.80	1.40	1.40	1.40	1.40
Chloride, g/kg	2.50	2.50	2.50	2.50	2.50	2.50
Potassium, g/kg	8.77	8.77	8.58	8.58	6.42	6.42
Magnesium, g/kg	1.61	1.61	1.61	1.61	1.63	1.63
Manganese, mg/kg	110 (90)	110 (88)	111 (81)	111 (96)	105 (72)	105 (87)
Zinc, mg/kg	108 (79)	108 (78)	109 (73)	109 (83)	111 (78)	111 (80)
Copper, mg/kg	20 (11)	20 (9)	21 (8)	21 (9)	21 (10)	21 (10)

^1^ Xylanase 3.2.1.8 and glucanase 3.2.1.6, Dupont, Wilmington, DE, USA. ^2^ There were two types of experimental premixes: oxide and sulfate and hydroxychloride, which were different only in the source of manganese, zinc and copper; the supplementary sources for oxide and sulfate: manganese oxide (62%), zinc oxide (72%), and copper sulfate (25%) and for hydroxychloride: Intellibond (Trouw Nutrition, Tilburg, The Netherlands). The supplemental levels of Mn, Zn, and Cu were 65, 50, and 5 mg/kg to the final feed, respectively. ^3^ Supplied 1000 FTU phytase, 10,000 IU/kg of vitamin A, 3000 IU/kg of vitamin D3, 30 IU/kg of vitamin E, 3 mg/kg of vitamin K3, 2 mg/kg of vitamin B1, 8 mg/kg of vitamin B2, 8 mg/kg of vitamin B2, 40 mg/kg of B3, 14 mg/kg of B5, 4 mg/kg of B6, 100 mcg/kg of B7, 1 mg/kg of B9, 25 mcg/kg of B12, 300 mg/kg of choline, 100 mg/kg of betaine, 25 mg/kg of iron, 1.9 mg/kg of iodine, and 0.15 mg/kg of selenium to the final diet. ^4^ The average of the samples and duplicated analysis. ^5^ Apparent fecal digestibility for all the amino acid values.

**Table 2:** The effect of different sources of manganese, zinc, and copper in rearing diets on growth performance in ISA Brown birds during the rearing phase.

Items	Oxide/Sulfate ^1^	Hydroxychloride ^2^	*p*-Value
Starter (1 to 3 weeks) ^3^			
Body weight at 3 weeks, g	221 ± 3.1	219 ± 3.1	0.615
Daily feed intake, g/bird/day	15.6 ± 0.33	15.6 ± 0.33	0.973
Feed conversion ratio	1.88 ± 0.012	1.90 ± 0.012	0.318
Grower (4 to 10 weeks) ^3^			
Body weight at 10 weeks, g	1131 ± 12.4	1152 ± 11.2	0.227
Daily feed intake, g/bird/day	56.0 ± 0.97	59.7 ± 0.94	0.004
Feed conversion ratio	3.02 ± 0.018	3.13 ± 0.016	0.002
Developer (11 to 16 weeks) ^3^			
Body weight at 16 weeks, g	1636 ± 22.5	1663 ± 20.3	0.355
Daily feed intake, g/bird/day	81.1 ± 2.55	83.1 ± 2.25	0.167
Feed conversion ratio	6.80 ± 0.205	6.84 ± 0.184	0.865
Overall (1 to 16 weeks) ^3^			
Daily feed intake, g/bird/day	54.3 ± 0.92	56.5 ± 0.85	0.059
Feed conversion ratio	3.803 ± 0.0298	3.891 ± 0.0280	0.023

^1^ Manganese, zinc, and copper sources for oxide/sulfate group were Mn-oxide (62%), Zn-oxide (72%), and Cu-sulfate (25%). The targeted supplementary Mn, Zn, and Cu levels were 65, 50, and 5 mg/kg of feed. ^2^ Manganese, zinc, and copper sources for hydroxychloride were Intellibond (Trouw Nutrition, Tilburg, The Netherlands). The targeted supplementary Mn, Zn, and Cu levels were 65, 50, and 5 mg/kg of feed. ^3^ Data are least-squares means of six replications and 10 birds each.

**Table 3:** The effect of different sources of manganese, zinc, and copper in rearing diets on egg production performance in ISA Brown birds up to 50 weeks of age.

Items	Oxide/Sulfate ^1^	Hydroxychloride ^2^	*p*-Value
18–24 weeks ^3^			
Daily feed intake, g	115.7 ± 1.73	114.6 ± 1.75	0.674
Hen-day egg production, %	97.91 ± 0.834	98.63 ± 0.851	0.570
Egg weight, g	55.4 ± 0.58	56.3 ± 0.58	0.270
Egg mass, g/d	54.24 ± 0.654	55.53 ± 0.662	0.172
Feed conversion ratio	2.149 ± 0.0436	2.107 ± 0.0438	0.501
Body weight at 25 weeks of age, g	1853 ± 30.3	1896 ± 30.3	0.322
25–37 weeks ^3^			
Daily feed intake, g	123.3 ± 2.18	129.4 ± 2.13	<0.001
Hen-day egg production, %	98.32 ± 0.256	99.07 ± 0.243	0.589
Egg weight, g	60.1 ± 0.70	62.4 ± 0.68	0.003
Egg mass, g/d	59.09 ± 0.770	61.82 ± 0.753	<0.001
Feed conversion ratio	2.102 ± 0.0269	2.098 ± 0.0257	0.880
38–50 weeks ^3^			
Daily feed intake, g	121.3 ± 2.47	128.7 ± 2.42	<0.001
Hen-day egg production, %	98.16 ± 0.404	96.70 ± 0.392	0.760
Egg weight, g	61.5 ± 0.81	64.2 ± 0.78	<0.001
Egg mass, g/d	60.37 ± 0.959	62.08 ± 0.931	0.076
Feed conversion ratio	2.019 ± 0.0582	2.145 ± 0.0557	0.081
Body weight at 50 weeks of age, g	2055 ± 56.0	2107 ± 52.1	0.502

^1^ Manganese, zinc, and copper sources for oxide/sulfate group were Mn-oxide (62%), Zn-oxide (72%), and Cu-sulfate (25%). The targeted supplementary Mn, Zn, and Cu levels were 65, 50, and 5 mg/kg of feed. ^2^ Manganese, zinc, and copper sources for hydroxychloride were Intellibond (Trouw Nutrition, Tilburg, The Netherlands). The targeted supplementary Mn, Zn, and Cu levels were 65, 50, and 5 mg/kg of feed. ^3^ Data are least-squares means of 18 replications (18 birds) per treatment.

**Table 4:** The effect of different sources of manganese, zinc, and copper in rearing diets on egg quality in ISA Brown birds.

Items	Oxide/Sulfate ^1^	Hydroxychloride ^2^	*p*-Value
25 to 37 weeks ^3^			
Albumin percentage	63.50 ± 0.367	64.62 ± 0.354	0.813
Yolk percentage	26.27 ± 0.317	25.48 ± 0.329	0.092
Eggshell percentage	10.23 ± 0.126	9.99 ± 0.128	0.206
Breaking strength, g	6131 ± 161.5	5935 ± 153.9	0.382
Eggshell thickness, mm	0.379 ± 0.0075	0.367 ± 0.0073	0.265
Shell weight per unit surface area	85.97 ± 0.999	84.89 ± 1.118	0.453
38 to 50 weeks ^3^			
Albumin percentage	63.05 ± 0.452	62.60 ± 0.468	0.191
Yolk percentage	26.91 ± 0.350	27.72 ± 0.354	0.116
Eggshell percentage	10.04 ± 0.120	9.68 ± 0.117	0.039
Breaking strength, g	5802 ± 206.1	5528 ± 203.0	0.346
Eggshell thickness, mm	0.376 ± 0.0066	0.372 ± 0.0067	0.685
Shell weight per unit surface area	85.13 ± 0.975	83.05 ± 0.954	0.138

^1^ Manganese, zinc, and copper sources for oxide/sulfate group were Mn-oxide (62%), Zn-oxide (72%), and Cu-sulfate (25%). The targeted supplementary Mn, Zn, and Cu levels were 65, 50, and 5 mg/kg of feed. ^2^ Manganese, zinc, and copper sources for hydroxychloride were Intellibond (Trouw Nutrition, Tilburg, The Netherlands). The targeted supplementary Mn, Zn, and Cu levels were 65, 50, and 5 mg/kg of feed. ^3^ Data are least-squares means of 18 replications (18 birds) per treatment with four eggs per cage collected every 4 weeks.

**Table 5:** The effect of different sources of manganese, zinc, and copper in rearing diets on tibia quality in ISA Brown birds.

Items	Oxide/Sulfate ^1^	Hydroxychloride ^2^	*p*-Value
16 weeks ^3^			
Dry weight, g	10.1 ± 0.20	10.3 ± 0.20	0.529
Ash content, g	3.3 ± 0.07	3.4 ± 0.07	0.186
Ash, %	32.3 ± 0.53	33.0 ± 0.53	0.311
Breaking strength, g	15,380 ± 432.4	14,652 ± 432.4	0.247
Cortical composition, % of DM			
Organic matter	23.0 ± 6.54	22.3 ± 6.54	0.637
Carbonate	27.8 ± 0.80	28.3 ± 0.80	0.678
Phosphate	49.1 ± 4.16	49.2 ± 4.16	0.914
50 weeks ^4^			
Dry weight, g	7.6 ± 0.28	9.0 ± 0.24	0.014
Ash content, g	3.9 ± 0.32	4.0 ± 0.32	0.908
Ash, %	46.6 ± 1.12	44.6 ± 1.12	0.252
Breaking strength, g	20,293 ± 2137.2	20,772 ± 2137.2	0.879
Cortical composition, % of DM			
Organic matter	25.6 ± 1.80	26.4 ± 1.38	0.600
Carbonate	41.0 ± 3.30	32.2 ± 3.30	0.132
Phosphate	33.9 ± 11.16	41.7 ± 8.05	0.347
Medullary composition, % of DM			
Organic matter	60.6 ± 6.25	59.0 ± 5.11	0.418
Carbonate	6.0 ± 1.02	5.1 ± 0.59	0.320
Phosphate	33.4 ± 9.24	35.9 ± 5.34	0.833

^1^ Manganese, zinc, and copper sources for oxide/sulfate group were Mn-oxide (62%), Zn-oxide (72%), and Cu-sulfate (25%). The targeted supplementary Mn, Zn, and Cu levels were 65, 50, and 5 mg/kg of feed. ^2^ Manganese, zinc, and copper sources for hydroxychloride were Intellibond (Trouw Nutrition, Tilburg, The Netherlands). The targeted supplementary Mn, Zn, and Cu levels were 65, 50, and 5 mg/kg of feed. ^3^ Data are least-squares means of six replications and one bird each. ^4^ Data are least-squares means of eight replications and one bird each.

## Data Availability

The datasets presented in this article are not readily available because the data are part of an ongoing study and due to technical limitations. Requests to access the datasets should be directed to R.A.M.K.
